# Prevalence and associated factors of glaucoma in the Russian Ural Eye and Medical Study

**DOI:** 10.1038/s41598-020-77344-z

**Published:** 2020-11-20

**Authors:** Mukharram M. Bikbov, Timur R. Gilmanshin, Rinat M. Zainullin, Gyulli M. Kazakbaeva, Inga I. Arslangareeva, Songhomitra Panda-Jonas, Renat I. Khikmatullin, Said K. Aminev, Ildar F. Nuriev, Artur F. Zaynetdinov, Yulia V. Uzianbaeva, Nikolay A. Nikitin, Svetlana R. Mukhamadieva, Dilya F. Yakupova, Ellina M. Rakhimova, Iulia A. Rusakova, Natalia I. Bolshakova, Kamila R. Safiullina, Jost B. Jonas

**Affiliations:** 1 Ufa Eye Research Institute, 90 Pushkin Street, Ufa, Bashkortostan Russia 450077; 2 Department of Ophthalmology, Medical Faculty Mannheim, Theodor-Kutzerufer 1, 68167 Mannheim, Germany

**Keywords:** Diseases, Health care

## Abstract

To assess the prevalence and associated factors of glaucoma in a Russian population. The population-based Ural Eye and Medical Study included 5899 (mean age 59.0 ± 10.7 years; range 40–94 years). Glaucomatous optic neuropathy was diagnosed using International Society of Geographical and Epidemiological Ophthalmology (ISGEO) criteria. Among 5545 participants with assessable optic disc photographs, 246 individuals [4.4%; 95% confidence interval (CI) 3.9, 5.0] had glaucoma, with open-angle glaucoma (OAG) in 177 individuals (3.2%; 95% CI 2.7, 3.7) and angle-closure glaucoma (ACG) in 69 individuals (1.2; 95% CI 1.0, 1.5), with IOP > 21 mmHg in 79 (32.1%) patients, and with 80 (32.5%) patients on glaucoma therapy. Glaucoma prevalence increased from 3/485 (0.6%; 95% CI 0.0, 1.3) in the age group of 40–45 years to 33/165 (20.0%; 95% CI 13.8, 26.2) in the group aged 80 + years. Higher OAG prevalence correlated with older age [odds ratio (OR) 1.07; 95% CI 1.04, 1.09; *P* < 0.001], longer axial length (OR 1.36; 95% CI 1.17, 1.58; *P* < 0.001), higher intraocular pressure (IOP) (OR 1.18; 95% CI 1.13, 1.23; *P* < 0.001), higher stage of lens pseudoexfoliation (OR 1.26; 95% CI 1.08, 1.47; *P* = 0.004) and lower diastolic blood pressure (OR 0.98; 95% CI 0.96, 0.99; *P* = 0.035). Higher ACG prevalence correlated with older age (OR 1.07; 95% CI 1.03, 1.11; *P* < 0.001), narrower anterior chamber angle (OR 0.81; 95% CI 0.77, 0.86; *P* < 0.001), and higher IOP (OR 1.30; 95% CI 1.23, 1.38; *P* < 0.001). Glaucoma caused moderate to severe vision impairment (MSVI) in 9 (4.9%; 95% CI 1.8, 8.1) out of 184 individuals with MSVI (OAG, n = 7; ACG, n = 2), and blindness in one (9.1%) of 11 blind individuals. In this population from Russia, two thirds of glaucoma patients were not on therapy, and in two thirds of the glaucoma patients IOP was ≤ 21 mmHg. Otherwise, glaucoma prevalence, OAG-to-ACG ratio, and glaucoma associations did not differ markedly from Caucasian and East Asian populations.

Together with age-related macular degeneration and myopic maculopathy, glaucomatous optic neuropathy belongs to the most common causes of irreversible moderate to severe visual impairment (MSVI) and irreversible blindness worldwide^1–3^. Meta-analyses of single population-based studies showed that for the year 2015 glaucoma caused globally 8.49% (80% uncertainty interval (UI) 2.99–15.66) of all cases of blindness and 19.0% of all cases of irreversible blindness, and 2.05% (80% UI: 0.62–4.03) of all cases with MSVI and 9.1% of all cases with irreversible MSVI^1,2^. In the period from 1990 and 2015, the number of individuals affected by glaucoma-related blindness worldwide increased from 2.5 million (80% UI: 0.3 million to 8.6 million) to 3.0 million (80% UI: 0.4 million to 9.9 million), and the number of people affected by glaucoma-related MSVI increased from 3.0 million (80% UI: 0.4 million to 9.9 million) to 4.0 million (80% UI: 0.6 million to 13.3 million)^1^.

Despite the importance of glaucoma as a leading cause of irreversible vision loss, and although Russia is one of the countries with the largest population and the country with by far the greatest surface area, there is no information available about the prevalence of glaucoma in Russia, the associations of glaucoma with ocular and general parameters in the Russian population, and the importance of glaucoma as cause for MSVI and blindness in Russia. By the same token, there have in general been conflicting reports about the correlation of glaucoma with other ocular parameters, such as myopia, axial length and central corneal thickness, and with systemic factors like diabetes mellitus and arterial blood pressure. We therefore conducted the present study to examine the prevalence of glaucoma and the frequency of glaucoma as cause for MSVI and blindness in a population from Russia, and to explore associations of glaucoma with other parameters. To reduce the potential bias caused by a referral of study participants, we chose a population-based recruitment of the study participants. To reduce the risk of a bias due to hidden confounding factors, we included a whole panoply of parameters and ocular and systemic diseases for the assessment of correlations between glaucoma and other factors.

## Methods

In the period from 2015 to 2017, the population-based Ural Eye and Medical Study (UEMS) was conducted in the city of Ufa in the district of Kirovskii and in villages in the rural region of the Karmaskalinsky District in a distance of 65 km from Ufa^4–6^. The Ethics Committee of the Academic Council of the Ufa Eye Research Institute approved the study design and confirmed that the study adhered to the Declaration of Helsinki, and all participants gave an informed written consent. Ufa is the capital of the Republic of Bashkortostan and is the economic, scientific and cultural center of the region. Situated at a distance of 1300 km East of Moscow in the West of the Southern Ural mountains, Ufa has 1.1 million inhabitants including Russians (49%), Tatars (28%), Bashkirs (17%), Ukrainians (1.2%) and other ethnicities. The hottest month is July with an average high temperature of 25.9 °C and the coldest months are January and February with an average low temperature of − 17 °C. All people residing in the study regions were officially registered, and home visits were performed according to the people registration. The eligible subjects fulfilling the inclusion criterion of an age of 40 + years were visited up to three times if they did not participate in the study after the first visit. The only inclusion criteria for the study were living in the study region and an age of 40 + years. There were no exclusion criteria.

The examinations started with an interview consisting of about 256 standardized questions on socioeconomic parameters such as level of education, family income and family possessions, living conditions (such as toilet available in the house, lighting source, agricultural land and livestock ownership, size of family), diet (such as frequency and amount of intake of vegetables, fruits and meat), smoking or other types of tobacco consumption, daily physical activity, alcohol consumption, presence of ocular problems, availability of an ophthalmologist, availability and wearing of glasses, depression and suicidal ideas, medical history including known diagnosis and therapy of arterial hypertension, diabetes mellitus, angina, asthma and other pulmonary problems, cardiovascular and cerebrovascular diseases, lower back pain, malignancies, menstruation and related issues, previous trauma including bone fractures, and hearing problems. The questions were taken from standardized questionnaires, such as the Zung self-rated depression scale and the Mini Mental Status Examination test^7,8^. The level of education was categorized into the stages of “illiteracy” (no reading ability at all), “passing of the 5th class”, “passing of the 8th class”, “passing of the 10th class”, “passing of the 11th class”, “graduation”, and “post-graduation”. The questions additionally included standardized questions on the amount and frequency of smoking and alcohol consumption and living conditions and were previously included and tested in other population-based studies such as the Central India Eye and Medical Study and the Beijing Eye Study^9,10^. The interview was conducted by trained social workers who personally asked the questions and filled the answers into the questionnaire.

Medical examinations included measurement of blood pressure, handgrip force and anthropometric parameters. We conducted a spirometric test for the assessment of chronic obstructive pulmonary disease, and a biochemical analysis of blood samples taken under fasting conditions. The ophthalmologic examinations included automatic and subjective refractometry for determination of best corrected visual acuity (BCVA), perimetry (PTS 1000 Perimeter, Optopol Technology Co., Zawercie, Poland), anterior segment biometry (Pentacam HR, Typ70900, OCULUS, Optikgeräte GmbH Co., Wetzlar, Germany), slit lamp biomicroscopy of the anterior ocular segment, and non-contact tonometry (Tonometer Kowa KT-800, Kowa Company Ltd., Hamamatsu City, Japan). The visual field test was repeated if there was an error as registered by the device or if the visual field did not correspond to the ophthalmological findings. In addition, unreliable perimetric results were excluded from the statistical analysis. The reliability of the visual field tests was assessed as the fixation errors, the falsely positive errors, and the falsely negative errors. The fixation errors were measured either by an automatic analysis of the fundus location or it assessed by the Heijl-Krakau method^11^. The fixation errors, false positive errors and the false negative errors had to be ≤ 25%. A visual field defect was defined as the presence of a cluster of three test points outside of the normal range.

After medically inducing mydriasis (tropicamide 0.8% and phenylephrine 5% given twice in a 10-min interval), a second slit lamp examination was performed by a board-certified ophthalmologist to assess the presence of pseudoexfoliation of the lens^12^. Pseudoexfoliation was differentiated into seven grades or stages, with stage 0 for “no pseudoexfoliation”, stage 1 for “faint pseudoexfoliation” (small dark islands in the intermediary annular region corresponding to the moving pupillary margin), stage 2 for “confluent dark islands in the annular region”, stage 3 for “visible edges of pseudoexfoliative material clearly detectable in at least one location on the lens surface”, stage 4 for “complete circular edge of pseudoexfoliative material on the lens surface (central island or in the lens periphery)”, stage 5 for “pseudoexfoliative dandruff on the pupil margin”, and stage 6 for “pseudoexfoliative material on the corneal endothelium, in the anterior chamber angle, and/or lens subluxation”. A similar grading classification had been described by Prince and associates^13^. Using lens photographs, the presence and degree of cataract was assessed by applying the scheme of the Age-Related Eye Disease Study^14^. We defined the presence of nuclear cataract as a nuclear cataract grade of 3 or higher. The degrees of cortical lens opacification and posterior subcapsular lens opacification were assessed using photographs taken by retro-illumination (Topcon slit lamp and camera, Topcon Corp. Tokyo, Japan). Cortical and posterior subcapsular opacities appear as darkly shaded interruptions of the reddish-orange fundus reflex on these photographs. Any lens area that is definitely darkened is considered involved, regardless of the density of the opacity. Using a grid, the degrees of cortical cataract and subcapsular cataract were measured as the percentages area of opacity. The presence of cortical cataract and subcapsular cataract was defined by the presence of any cortical or subcapsular opacity, respectively. We additionally took photographs of the optic nerve head and macula (VISUCAM 500, Carl Zeiss Meditec AG, Jena, Germany) and spectral-domain optical coherence tomographic (OCT) images (RS-3000, NIDEK co., Ltd., Aichi Japan). The latter served for measurement of the peripapillary retinal nerve fiber layer thickness, neuroretinal rim width, and thickness of the retina. The degree of fundus tessellation was examined on the fundus photographs^15^. We defined age-related macular degeneration (AMD) as suggested by the recent Beckman Initiative for Macular Research Classification Committee^16^. For the definition of glaucoma we applied criteria recommended by the ISGEO (International Society of Geographical and Epidemiological Ophthalmology)^17^. The anterior segment images taken with the Pentacam camera were used to differentiate between open-angle glaucoma and angle-closure glaucoma. The anterior chamber angle was considered to be closed if the peripheral iris had direct contact with the peripheral cornea.

We defined diabetes mellitus by a glucose concentration of ≥ 7.0 mmol/L or a self-reported history of a physician-based diagnosis of diabetes mellitus or a history of drug treatment for diabetes. Arterial hypertension was defined using the criteria defined by the American College of Cardiology/American Heart Association^18^. All examinations and the interview were conducted in the Ufa Eye Research Institute in Ufa.

Out of 7328 eligible individuals, the study population consisted of 5899 individuals (2580 (43.7%) men) with a mean age of 59.0 ± 10.7 years (range: 40–94 years) who had agreed to participate. The participation rate was 80.5%. The study population did not differ significantly in the gender and age distribution from the Russian population as explored in the census carried out in 2010^19^. Stratified by ethnicity, the study population included 1185 (20.1%) Russians, 2439 (41.3%) Tartars, 1061 (18.0%) Bashkirs, 587 (10%) Chuvash, 21 (0.4%) Mari, 104 (1.8%) individuals of other ethnicities, and 502 (8.5%) individuals did not indicate their ethnic background. The proportion of non-Russians on the total study population was higher than the proportion of non-Russians on the total population of Russia.

For the present study, we included all participants for whom information about the presence of glaucoma was available. Applying a software package (SPSS/Windows, 25.0, IBM-SPSS, Chicago, IL, USA), we first calculated mean values (and 95% confidence intervals (CI)) of the prevalence of glaucoma as a whole and separated into open-angle glaucoma and angle-closure glaucoma^4,5^. In a following binary univariate regression analyses, we tested relationships between the prevalence of the glaucomas and systemic and ocular parameters. We eventually performed a multivariable binary regression analysis to assess associations between the glaucoma prevalence and all those variables which were significantly (*P* ≤ 0.05) correlated with the glaucoma prevalence in the univariate analyses. Odds ratios (OR) and their 95% CI were determined.

## Results

Information about the presence of glaucoma was available for 5545 (94.0%) individuals with a mean age of 58.5 ± 10.5 years (range 40–94 years) and a mean axial length of 23.3 ± 1.1 mm (range 19.78–32.87 mm) (Table 1). The group of participants with information about glaucoma as compared with the group of individuals without assessment of the presence of glaucoma was significantly (*P* < 0.001) younger (58.5 ± 10.5 years versus 65.8 ± 11.5 years) and showed a significantly (*P* < 0.001) higher proportion of women (3152/5545 or 56.8% versus 167/354 or 47.2%), while both groups did not differ significantly (*P* = 0.45) in axial length (23.3 ± 1.1 mm versus 23.3 ± 1.3 mm).

**Table 1:** Demographic data (mean ± standard deviation) of the study population.

Parameter		
Age	Years	58.5 ± 10.5
Gender	Men/women	2393 (43.2%)/3152 (56.8%
Region of habitation	Urban/rural	2262 (40.8%)/3283 (59.2%)
Ethnicity	Russian	1111 (20.0%)
Body height	cm	164.8 ± 8.7
Body weight	kg	76.0 ± 14.5
Body mass index	kg/m^2^	28.0 ± 5.0
Waist circumference	cm	94.0 ± 13.3
Hip circumference	cm	103.7 ± 12.5
Waist/hip circumference ratio	Ratio	0.91 ± 0.09
Family status	Joint (three generations)/nuclear (parents and children)/two people/single	1452 (26.2%)/2336 (42.1%)/1439 (26.0%)/302 (5.4%) (missing: 16 (0.3%)
Family status	Married/unmarried/divorced/widowed	4093 (73.8%)/343 (6.2%)/312 (5.6%)/796 (14.4%) (Missing: 1 (0.01%)
Religion	Muslim/christian/others	3491 (62.8%)/1977 (35.7%)/87 (1.6%)
Level of education	Illiteracy/passing 5th grade/8th grade/10th grade/11th grade/specialized secondary education/graduates/post graduates	11 (0.2%)/86 (1.6%)/545 (9.8%)/619 (11.2%)/727 (13.1%)/1584 (28.6%)/1922 (34.7%)/50 (0.9%) (Missing: 1 (0.01%)

All study participants had fundus photographs for the assessment of the optic nerve head, while about 20% of all eyes with an advanced stage of glaucomatous optic neuropathy, as indicated by a vertical cup/disc diameter ratio of ≥ 0.9 or an inter-eye asymmetry in the vertical cup/disc diameter ratio of ≥ 0.3, did not have reliable visual field tests. Measurements of the peripapillary retinal nerve fiber layer thickness were available for 5040 /5545 or 90.9% of the study participants, and photographs of the lens were available for 4904/5545 (88.4%) of the study participants.

**Figure 1:**
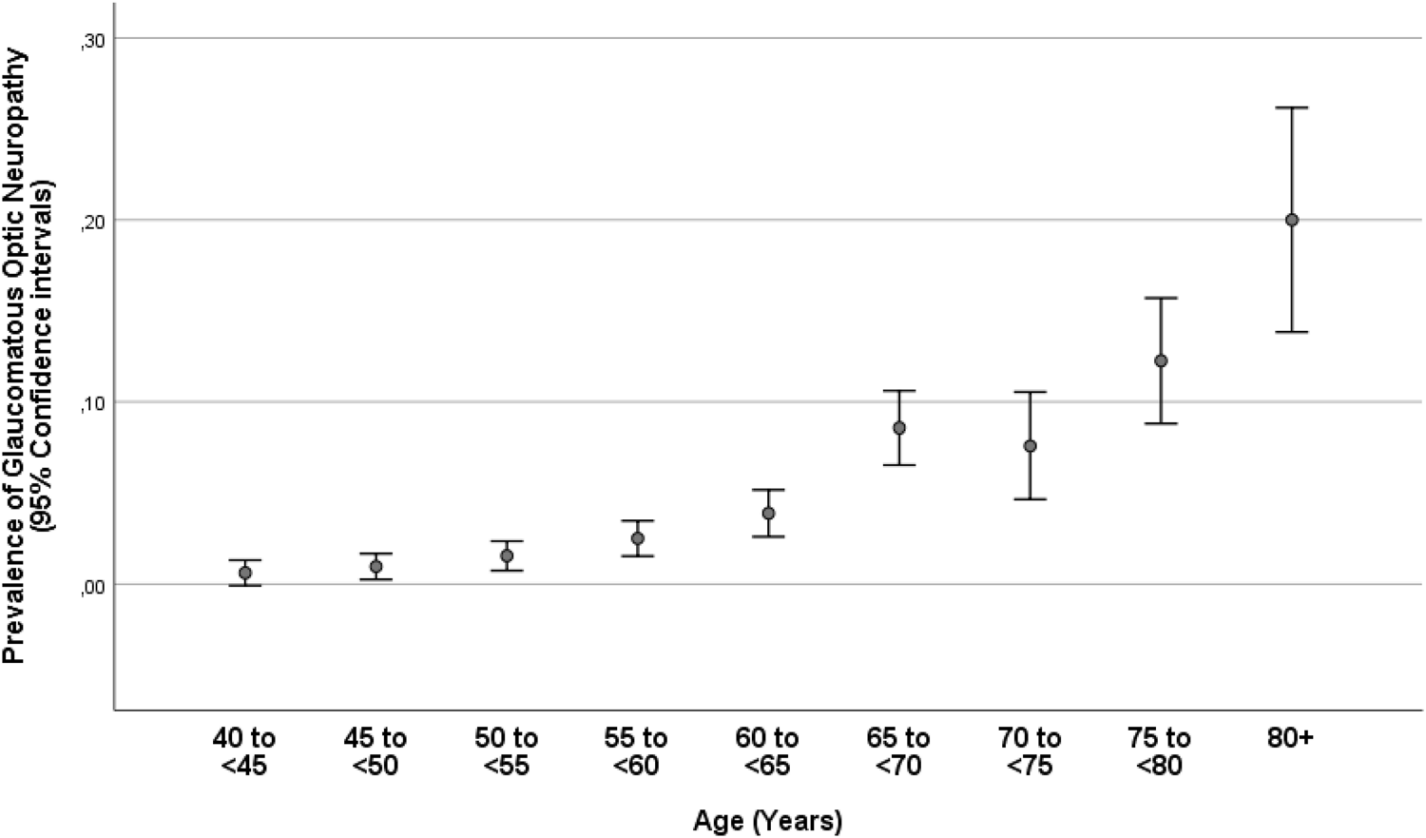
Prevalence of glaucomatous optic neuropathy in the Ural Eye and Medical Study.

**Figure 2:**
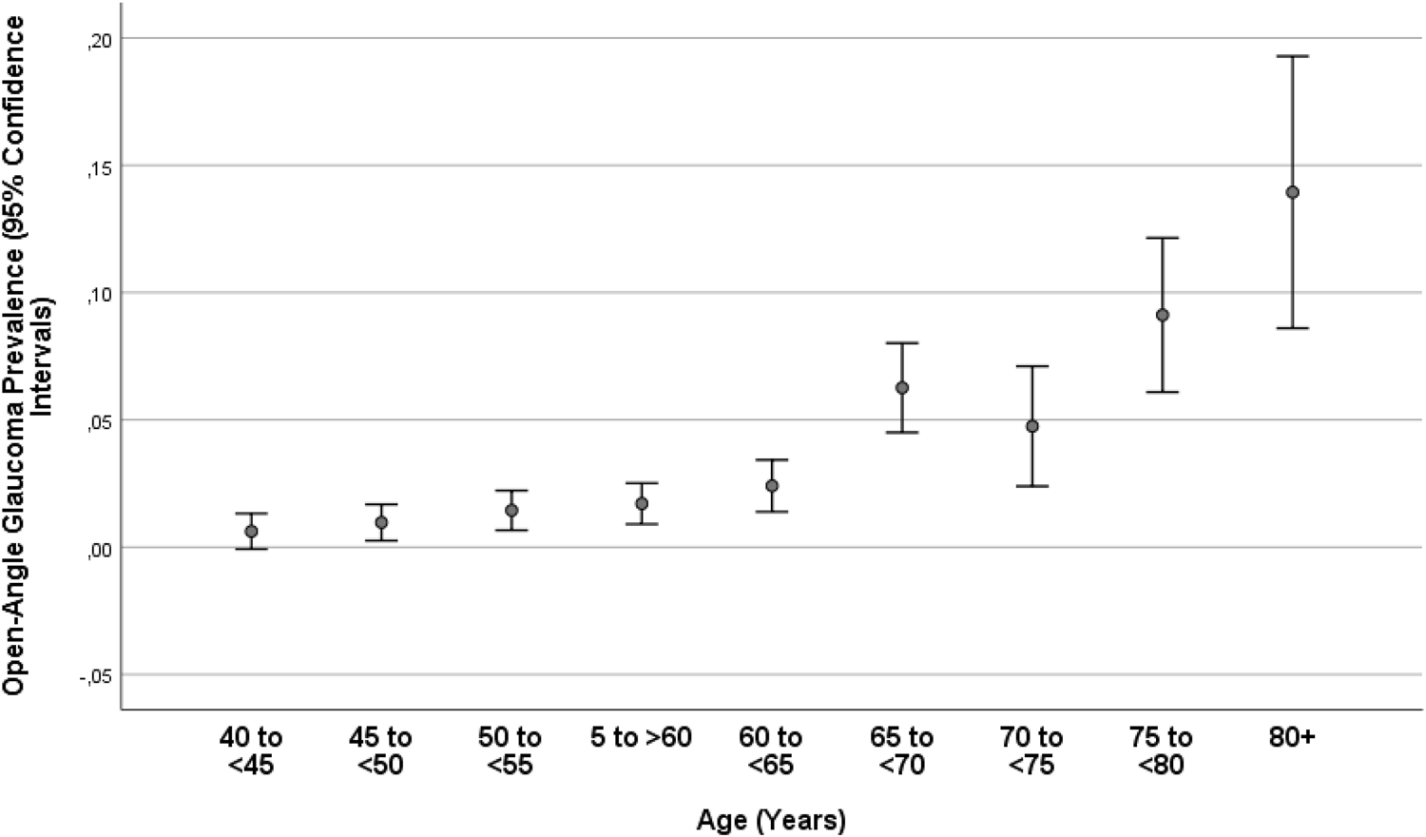
Prevalence of open-angle glaucoma in the Ural Eye and Medical Study.

**Figure 3:**
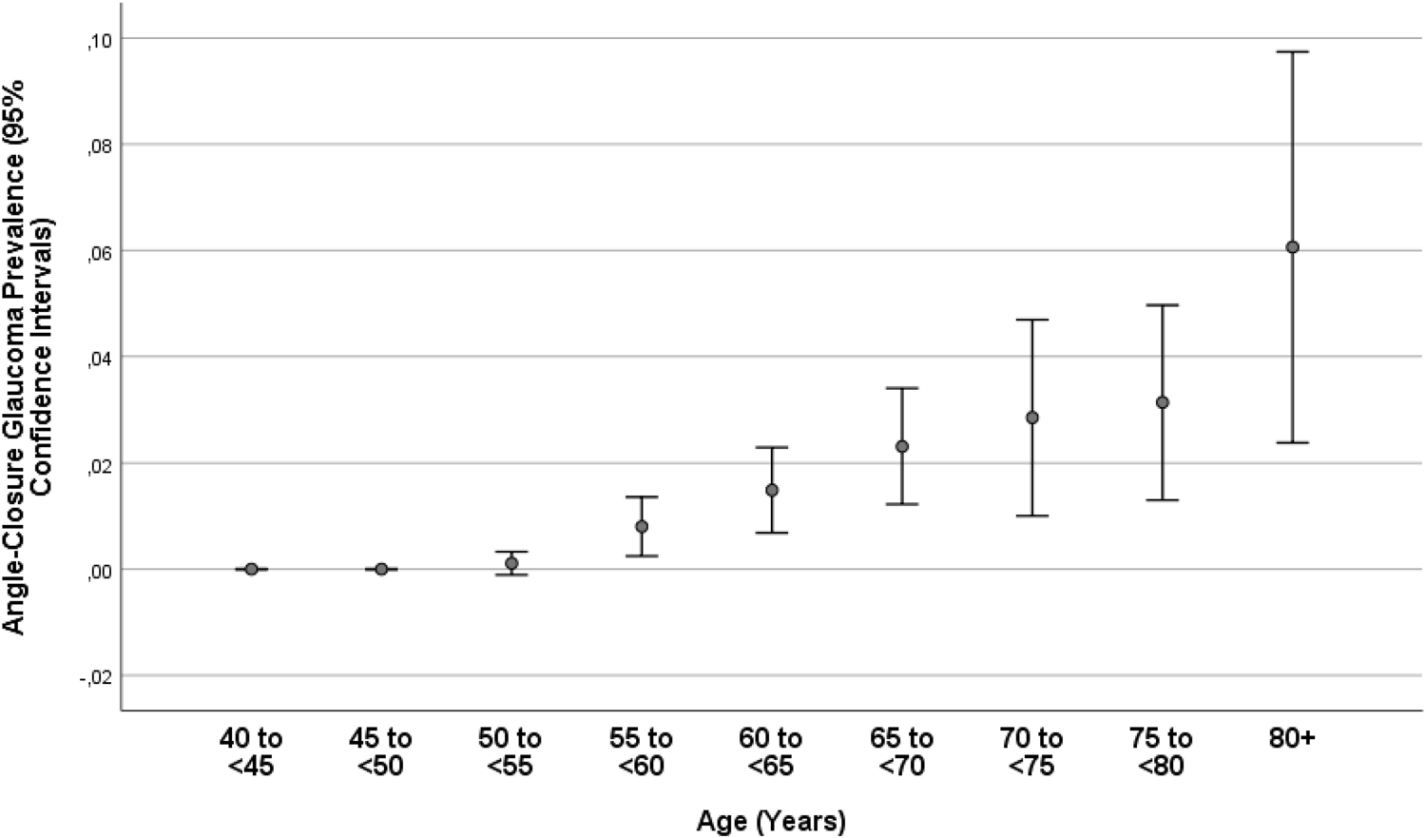
Prevalence of angle-closure glaucoma in the Ural Eye and Medical Study.

Glaucoma in any eye of a participant was present in 246/5545 individuals (4.4%; 95% CI 3.9, 5.0), with open-angle glaucoma being prevalent in 177/5545 individuals (3.2%; 95% CI 2.7, 3.7) and angle-closure glaucoma in 69/5545 individuals (1.2%; 95% CI 1.0, 1.5). Among the individuals with open-angle glaucoma, 25/177 (14.1%) had secondary glaucoma due to pseudoexfoliation, and among the individuals with angle-closure glaucoma, 7/69 (10.1%) had pseudoexfoliation. The prevalence of glaucoma increased from 3/485 (0.6%; 95% CI 0.0, 1.3) in the age group of 40–< 45 years to 34/873 (3.9%; 95% CI 2.6, 5.2) in the age group of 60–< 65 years, and to 33/165 (20.0%; 95% CI 13.8, 26.2) in the age group of 80 + years (Table 2; Fig. 1). The prevalence of open-angle glaucoma increased from 3/485 (0.6%; 95% CI 0.0, 1.3) in the age group of 40–< 45 years to 21/873 (2.4%; 95% CI 1.4, 3.4) in the age group of 60–< 65 years, and to 23/165 (13.9%; 95% CI 8.6, 19.3) in the age group of 80 + years (Fig. 2). The prevalence of angle-closure glaucoma increased from 0/485 (0.0%) in the age group of 40–< 45 years to 13/873 (1.5%; 95% CI 0.7, 2.3) in the age group of 60–< 65 years, and to 10/165 (6.1%; 95% CI 2.4, 9.7) in the age group of 80 + years (Table 2; Fig. 3). Among the 246 patients with glaucoma, 80 (32.5%) patients were on glaucoma therapy. IOP was higher than 21 mmHg in 79 (32.1%; 95% CI 26.4, 38.2) patients.

**Table 2:** Prevalence (number and percentage) of open-angle glaucoma and angle-closure glaucoma, stratified by age, in the Ural Eye and Medical Study.

Age group	n	Open-angle glaucoma	Angle-closure glaucoma
40–< 45 years	485	3 (0.6%)	0 (0%)
45–< 50 years	724	7 (1.0%)	0 (0%)
50–< 55 years	901	13 (1.4%)	1 (0.1%)
55–< 60 years	995	17 (1.7%)	8 (0.8%)
60–< 65 years	873	21 (2.4%)	13 (1.5%)
65–< 70 years	735	46 (6.3%)	17 (2.3%)
70–< 75 years	316	16 (5.1%)	9 (2.8%)
75–< 80 years	351	31 (8.8%)	11 (3.1%)
80 + years	165	23 (13.9%)	10 (6.1%)
Total	5545	178 (3.2%)	69 (1.2%)

After adjusting for age, a higher prevalence of open-angle glaucoma was associated with the systemic parameters of urban region of habitation (*P* = 0.04) and the prevalence of previous falls (*P* < 0.001), and with the ocular parameters of longer axial length (*P* < 0.001), more myopic refractive error (*P* < 0.001), deeper anterior chamber depth (*P* < 0.001), larger anterior chamber volume (*P* < 0.001), wider anterior chamber angle (*P* < 0.001), lower retinal nerve fiber layer thickness (*P* < 0.001), higher prevalence (*P* = 0.003) and grade (*P* = 0.001) of pseudoexfoliation of the lens, higher intraocular pressure (IOP) (*P* < 0.001), higher prevalence (*P* < 0.001) and stage (*P* < 0.001) of myopic maculopathy, and lower BCVA (*P* < 0.001) (Tables 3, 4).

**Table 3:** Associations (binary univariate analysis) between the prevalence of open-angle glaucoma and systemic parameters after adjusting for age in the Ural Eye and Medical Study.

Parameter	Interval	Odds ratio (OR)	95% confidence interval of OR	*P* value
Gender	Men/women	0.74	0.53, 1.04	0.08
Region of habitation	Urban/rural	1.44	1.02, 2.03	0.04
Ethnicity	Non-Russian ethnicity/Russian	1.28	0.87, 1.90	0.21
Body height	1 cm	1.02	0.998, 1.04	0.07
Body weight	kg	1.001	0.99, 1.01	0.89
Body mass index	kg/m^2^	0.99	0.95, 1.02	0.39
Waist circumference	cm	0.996	0.98, 1.01	0.53
Hip circumference	cm	1.001	0.99, 1.01	0.83
Waist/hip circumference ratio	Ratio	0.29	0.04, 2.12	0.22
Family status	Married versus unmarried	1.16	0.80, 1.67	0.44
Religion	Muslim versus non-Muslim	0.83	0.59, 1.16	0.28
Socioeconomic score	Score	0.97	0.88, 1.07	0.50
Level of education	Illiteracy/passing 5th grade/8th grade/10th grade/11th Grade/graduates/specialized secondary education/post graduates	0.97	0.88, 1.08	0.61
Physical activity score	Score	0.98	0.95, 1.01	0.17
Smoking , currently	Yes/no	0.92	0.47, 1.78	0.80
Smoking, package years	Number	1.00	0.99, 1.02	0.59
Smoking, daily	Yes/no	1.06	0.56, 2.00	0.86
Alcohol consumption, any	Yes/no	1.41	0.92, 2.15	0.11
Number of meals taken	Number	0.91	0.74, 1.13	0.40
In a week how many days do you eat fruits?	Number of days	0.97	0.89, 1.05	0.40
In a week how many days do you eat vegetables?	Number of days	0.98	0.88, 1.09	0.67
Type of oil used for cooking	Vegetarian oil/non-vegetarian oil	0.94	0.46, 1.91	0.85
Food contained whole grain	Yes/no	0.97	0.64, 1.48	0.88
Amount of self-reported salt intake	Gram	1.01	0.94, 1.08	0.83
Grade of processing of meat	(Weak/medium/well done)	0.86	0.62, 1.19	0.36
History of cardiovascular disorders including stroke	Yes/no	1.37	0.95, 1.96	0.09
History of angina pectoris	Yes/no	0.80	0,40, 1.59	0.53
History of asthma	Yes/no	1.15	0.49, 2.69	0.74
History of arthritis	Yes/no	0.81	0.56, 1.17	0.27
History of previous bone fractures	Yes/no	0.70	0.47, 1.06	0.09
History of injuries other than bone fractures	Yes/no	0.98	0.35, 2.92	0.97
History of low back pain	Yes/no	1.42	0.99, 2.04	0.06
History of thoracic spine pain	Yes/no	1.17	0.78, 1.76	0.44
History of neck pain	Yes/no	1.20	0.82, 1.76	0.35
History of headache	Yes/no	1.20	0.84, 1.70	0.32
History of cancer	Yes/no	1.33	0.63, 2.79	0.46
History of dementia	Yes/no	0.00	0,.00	1.00
History of diarrhea	Yes/no	1.70	0.22, 13.1	0.61
History of iron-deficiency anemia	Yes/no	1.51	0.72, 3.17	0.27
History of low blood pressure episode and hospital admittance	Yes/no	0.74	0.30, 1.84	0.51
History of osteoarthritis	Yes/no	1.13	0.74, 1.74	0.57
History of skin disease	Yes/no	0.55	0.20, 1.50	0.24
History of thyroid disorder	Yes/no	0.72	0.39, 1.31	0.28
History of falls	Yes/no	0.57	0.35, 0.92	0.001
History of unconsciousness	Yes/no	0.93	0.52, 1.68	0.81
Age of the last menstrual bleeding	Years	1.00	0.95, 1.05	0.92
Age of last regular menstrual bleeding	Years	1.00	0.95, 1.05	0.92
History of menopause	Yes/no	1.21	0.34, 4.30	0.77
**Serum concentration of**				
Alanine aminotransferase	IU/L	1.00	0.99, 1.01	0.94
Aspartate aminotransferase	IU/L	1.00	0.98, 1.01	0.55
Aspartate aminotransferase-to- Alanine aminotransferase ratio	Ratio	1.06	0.81, 1.39	0.66
Bilirubin, total	µmol/L	1.00	0.99, 1.02	0.64
High-density lipoproteins	mmol/L	0.93	0.76, 1.15	0.49
Low-density lipoproteins	mmol/L	0.96	0.82, 1.12	0.63
Cholesterol	mmol/L	0.98	0.87, 1.09	0.66
Triglycerides	mmol/L	0.93	0.70, 1.22	0.58
Rheumatoid factor	IU/mL	0.95	0.81, 1.12	0.54
Erythrocyte sedimentation rate	mm/min	1.00	0.98, 1.01	0.62
Glucose	mmol/L	1.04	0.96, 1.13	0.37
Urea	mmol/L	1.10	0.90, 1.13	0.88
Creatinine	µmol/L	1.00	0.99, 1.01	0.87
Prothrombin time	INR (international normalization ratio)	0.98	0.29, 3.30	0.97
Hemoglobin	g/L	1.00	0.99, 1.01	0.66
Erythrocyte count	10^6^ cells/µL	0.94	0.60, 1.48	0.79
Leukocyte count	10^9^ cells/L	0.95	0.84, 1.07	0.36
Prevalence of diabetes mellitus	Yes/no	1.25	0.80, 1.94	0.32
Estimated glomerular filtration rate	30 mL/min/1.73 m^2^	1.01	0.99, 1.01	0.40
Stage of chronic kidney disease	0–5	0.99	0.96, 1.01	0.31
Anemia	Yes/no	1.10	0.76, 1.60	0.61
Blood pressure, systolic (SBP)	mmHg	1.00	0.99, 1.01	0.94
Blood pressure, diastolic (DBP)	mmHg	0.99	0.97, 1.01	0.18
Blood pressure, mean	mmHg	1.00	0.98, 1.01	0.48
Ankle-brachial index	Index	1.008	0.29, 3.94	0.91
Arterial hypertension	Yes/no	1.14	0.80, 1.32	0.48
Arterial hypertension, stage	0–4	1.07	0.90, 1.28	0.45
Prevalence of chronic obstructive pulmonary disease	Yes/no	0.75	0.34, 1.63	0.46
Intake of blood lipid lowering medication	Yes/no	1.14	0.65, 1.98	0.65
Hearing loss	Hearing loss score (0–44)	1.00	0.98, 1.01	0.52
Depression Score	Depression score unit (range: − 4 to + 15)	1.03	0.99, 1.08	0.20
State-Trait Anxiety Inventory	State-Trait Anxiety Inventory Score (range: − 7 to 13)	1.03	0.98, 1.08	0.28
Manual dynamometry, right hand	dekaNewton	1.00	0.98, 1.02	0.92
Manual dynamometry, right hand	dekaNewton	1.00	0.98, 1.02	0.91

**Table 4:** Associations (binary univariate analysis) between the prevalence of open-angle glaucoma and ocular parameters after adjusting for age in the Ural Eye and Medical Study.

Parameter	Interval	Odds ratio (OR)	95% confidence interval of OR	*P* value
Best corrected visual acuity	logMAR	1.65	1.28, 2.13	< 0.001
Refractive error, spherical equivalent	Diopters	0.86	0.82, 0.91	< 0.001
Refractive error, cylindrical value	Diopters	0.84	0.71, 1.003	0.054
Axial length	mm	1.60	1.42, 1.81	< 0.001
Corneal refractive power	Diopters	0.95	0.86, 1.06	0.40
Central corneal thickness	µm	1.001	0.996, 1.01	0.75
Corneal volume	mm^3^	0.99	0.95, 1.03	0.58
Anterior chamber depth	mm	2.33	1.87, 2.90	< 0.001
Anterior chamber volume	µL	1.02	1.02, 1.02	< 0.001
Anterior chamber angle	Degree	1.07	1.05, 1.09	< 0.001
Lens thickness	mm	0.71	0.46, 1.11	0.13
Myopic maculopathy	Yes/no	4.53	2.03, 10.1	< 0.001
Myopic maculopathy	Stage	2.02	1.56, 2.61	< 0.001
Nuclear cataract degree	Grade	1.24	0.99, 1.56	0.07
Nuclear cataract, presence	Yes/no	1.24	0.78, 1.97	0.36
Cortical cataract, degree	Percentage	0.99	0.97, 1.01	0.37
Cortical cataract, presence	Yes/no	1.03	0.61, 1.74	0.91
Subcapsular cataract, degree	Percentage	1.02	0.96, 1.08	0.60
Subcapsular cataract, presence	Yes/no	1.47	0.19, 11.2	0.71
Fundus tessellation, macula region	Grade	1.40	1.16, 1.69	0.001
Fundus tessellation, peripapillary region	Grade	1.47	1.22, 1.76	< 0.001
Intraocular pressure (mmHg)	mmHg	1.21	1.17, 1.24	< 0.001
Pseudoexfoliation of the lens, presence	Yes/no	2.31	1.34, 3.96	0.003
Pseudoexfoliation of the lens, grade	Grade	1.26	1.10, 1.45	0.001
Retinal thickness (total), fovea	µm	1.001	0.999, 1.004	0.31
Retinal thickness (total), 300 µm temporal to the fovea	µm	1.000	0.996, 1.004	0.91
Retinal thickness (total), 300 µm nasal to the fovea	µm	1.002	1.000, 1.005	0.10
Retinal nerve fiber layer thickness	µm	0.96	0.95, 0.97	< 0.001
Diabetic retinopathy	Yes/no	1.22	0.37, 3.96	0.74
Diabetic retinopathy, ETDRS grading	Scale	1.01	0.97, 1.05	0.72

The prevalence of open-angle glaucoma was not significantly associated with the systemic parameters of gender, ethnicity (Russian versus non-Russian, level of education, physical activity score, family status (married versus unmarried, religion (Muslim versus non-Muslim, body height, body weight, body mass index, waist-hip ratio, and history of angina pectoris, arthritis, bone fractures, cancer, cardiovascular diseases including stroke, dementia, history of diarrhea, heart attack, iron-deficiency anemia, episodes with low blood pressure and hospitalization, osteoarthritis, injuries other than bone fracture, backache, headache, neck pain, thoracic spine pain, skin disease, steroid (cortisone) therapy, thyreopathy or the history of episodes of unconsciousness. It was neither associated with data on menopause (age of any last bleeding, age of last regular bleeding), serum concentrations of alanine aminotransferase, aspartate aminotransferase, bilirubin, high-density lipoproteins, low-density lipoproteins, triglycerides, cholesterol, rheumatoid factor, erythrocyte sedimentation rate, glucose, creatinine, urea, residual nitrogen, and hemoglobin, prothrombin time, the erythrocyte and leucocyte count, the intake of blood lipid lowering medication, the prevalences of diabetes mellitus, anemia and arterial hypertension, the stage of arterial hypertension, State-Trait Anxiety Inventory (STAI) score, diet parameters such number of meals taken, number of days with fruit intake or vegetable intake, type of oil used for cooking, amount of food contained whole grain, amount of self-reported salt intake, and the grade of processing of meat (weak/medium/well done), systolic, diastolic and mean blood pressure, ankle-brachial index, right side, prevalence of arterial hypertension, current smoker status, daily smoking, smoked package years, any alcohol consumed, hearing loss total score, depression score, and manual dynamometry. Nor was the prevalence of open-angle glaucoma associated with the ocular parameters of corneal refractive power, central corneal thickness, lens thickness, retinal thickness (total) in the fovea, prevalence and stage of nuclear cataract, cortical cataract and subcapsular cataract, and prevalence and stage of diabetic retinopathy.

In the multivariable analysis, we dropped due to collinearity the parameters of prevalence of pseudoexfoliation (variance inflation factor (VIF): 12.5), anterior chamber angle and volume (VIF: 4.3), anterior chamber depth (VIF: 2.1), and prevalence of myopic maculopathy (VIF: 2.1). Due to a lack of statistical significance, we dropped the parameters of BCVA (*P* = 0.55), refractive error (*P* = 0.26), region of habitation (*P* = 0.16), stages of myopic maculopathy (*P* = 0.08). In the final model, a higher prevalence of open-angle glaucoma was associated with older age (OR 1.07; 95% CI 1.04, 1.09; *P* < 0.001), longer axial length (OR 1.36; 95% CI 1.17, 1.58; *P* < 0.001), higher IOP (OR 1.18; 95% CI 1.13, 1.23; *P* < 0.001), stage of lens pseudoexfoliation (OR 1.26; 95% CI 1.08, 1.47; *P* = 0.004), lower diastolic blood pressure (OR 0.98; 95% CI 0.96, 0.99; *P* = 0.035), and thinner peripapillary retinal nerve fiber layer thickness (OR 0.97; 95% CI 0.96, 0.98; *P* < 0.001).

After adjusting for age, a higher prevalence of angle-closure glaucoma was associated with the systemic parameters of ethnicity (Russian versus non-Russian; *P* = 0.04), smaller waist circumference (*P* = 0.03) and hip circumference (*P* = 0.005), higher prevalence of previous bone fractures (*P* = 0.04), lower erythrocyte sedimentation rate (*P* = 0.04), and with the ocular parameters of shorter axial length (*P* = 0.07), shallower anterior chamber depth (*P* < 0.001), smaller anterior chamber volume (*P* < 0.001), narrower anterior chamber angle (*P* < 0.001), thicker central corneal thickness (*P* = 0.001), thicker lens thickness (*P* = 0.02), higher intraocular pressure (*P* < 0.001), and lower retinal nerve fiber layer thickness (*P* < 0.001) (Tables 5, 6).

**Table 5:** Associations (binary univariate analysis) between the prevalence of angle-closure glaucoma and systemic parameters after adjusting for age in the Ural Eye and Medical Study.

Parameter	Interval	Odds ratio (OR)	95% confidence interval of OR	*P* value
Gender	Men/women	0.77	0.47, 1.27	0.31
Region of habitation	Urban/Rural	1.08	0.65, 1.80	0.77
Ethnicity	Non-Russian ethnicity/Russian	1.73	1.02, 2.96	0.04
Body height	1 cm	0.99	0.96, 1.02	0.49
Body weight	kg	0.99	0.98, 1.02	0.52
Body mass index	kg/m^2^	0.99	0.94, 1.05	0.81
Waist circumference	cm	0.98	0.96, 0.997	0.03
Hip circumference	cm	0.97	0.96, 0.99	0.005
Waist/hip circumference ratio	Ratio	2.55	0.17, 37.7	0.50
Family status	Married versus unmarried	0.82	0.48, 1.40	0.47
Religion	Muslim versus non-Muslim	0.76	0.46, 1.26	0.29
Socioeconomic score	Score	0.97	0.84, 1.13	0.72
Level of education	Illiteracy/passing 5th grade/8th grade/10th grade/11th grade/graduates/specialized secondary education/post graduates	1.00	0.86, 1,17	0.97
Physical activity Score	Score	1.01	0.97, 1.05	0.64
Smoking , currently	Yes/no	1.74	0.77, 3.96	0.19
Smoking, package years	Number	1.00	0.98, 1.03	0.69
Smoking, daily	Yes/no	1.49	0.62, 3.57	0.37
Alcohol consumption, any	Yes/no	1.81	0.99, 3.31	0.06
Number of meals taken	Number	0.99	0.72, 1.35	0.93
In a week how many days do you eat fruits?	Number of days	1.01	0.89, 1.14	0.90
In a week how many days do you eat vegetables?	Number of days	1.05	0.88, 1.26	0.60
Type of oil used for cooking	Vegetarian oil/non-vegetarian oil	0.77	0.10, 5.71	0.80
Food contained whole grain	Yes/no	1.37	0.70, 2.66	0.36
Amount of self-reported salt intake	Gram	1.07	0.98, 1.16	0.14
Grade of processing of meat	(Weak/medium/well done)	1.36	0.81, 2.28	0.24
History of cardiovascular disorders including stroke	Yes/no	0.81	0.47, 1.41	0.46
History of angina pectoris	Yes/no	0.63	0.19, 2.02	0.43
History of asthma	Yes/no	1.34	0.41, 4.36	0.63
History of arthritis	Yes/no	0.91	0.53, 1.57	0.74
History of previous bone fractures	Yes/no	1.70	1.02, 2.84	0.04
History of injuries other than bone fractures	Yes/no	0.51	0.07, 3.76	0.51
History of low back pain	Yes/no	0.77	0.46, 1.28	0.32
History of thoracic spine pain	Yes/no	0.68	0.34, 1.34	0.26
History of neck pain	Yes/no	0.70	0.38, 1.31	0.27
History of headache	Yes/no	0.62	0.37, 1.06	0.08
History of cancer	Yes/no	0.72	0.17, 3.00	0.65
History of dementia	Yes/no	1.05	0.14, 8.12	0.96
History of diarrhea	Yes/no	0.00	0.00	1.00
History of iron-deficiency anemia	Yes/no	1.23	0.38, 4.00	0.73
History of low blood pressure episode and hospital admittance	Yes/no	1.43	0.51, 4.04	0.50
History of osteoarthritis	Yes/no	0.81	0.41, 1.60	0.54
History of skin disease	Yes/no	0.60	0.14, 2.47	0.48
History of thyroid disorder	Yes/no	1.39	0.68, 2.85	0.37
History of falls	Yes/no	0.96	0.52, 1.78	0.90
History of unconsciousness	Yes/no	1.85	0.93, 3.69	0.08
Age of the last menstrual bleeding	Years	0.99	0.93, 1.07	0.87
Age of last regular menstrual bleeding	Years	0.99	0.92, 1.06	0.77
History of menopause	Yes/no	1.25	0.15, 10.5	0.84
**Serum concentration of**				
Alanine aminotransferase	IU/L	1.00	0.98, 1.02	0.89
Aspartate aminotransferase	IU/L	1.01	0.99, 1.02	0.48
Aspartate aminotransferase-to- Alanine aminotransferase ratio	Ratio	1.16	0.90, 1.49	0.25
Bilirubin, total	µmol/L	1.01	0.99, 1.03	0.57
High-density lipoproteins	mmol/L	0.80	0.58, 1.11	0.19
Low-density lipoproteins	mmol/L	0.93	0.73, 1.17	0.53
Cholesterol	mmol/L	0.83	0.68, 1.02	0.07
Triglycerides	mmol/L	0.79	0.50, 1.25	0.32
Rheumatoid factor	IU/mL	0.97	0.77, 1.22	0.78
Erythrocyte sedimentation rate	mm/min	0.97	0.95, 0.998	0.04
Glucose	mmol/L	1.04	0.92, 1.18	0.53
Urea	mmol/L	0.83	0.68, 1.02	0.07
Creatinine	µmol/L	1.00	0.99, 1.01	0.80
Prothrombin time	INR (international normalization ratio)	0.37	0.05, 2.61	0.32
Hemoglobin	g/L	1.00	0.98, 1.02	0.65
Erythrocyte count	10^6^ cells/µL	1.54	0.77, 3.09	0.23
Leukocyte count	10^9^ cells/L	1.10	0.95, 1.28	0.22
Prevalence of diabetes mellitus	Yes/no	1.10	0.55, 2.19	0.79
Estimated glomerular filtration rate	30 mL/min/1.73m^2^	1.00	0.98, 1.02	0.94
Stage of chronic kidney disease	0–5	1.01	0.97, 1.05	0.67
Anemia	Yes/no	1.33	0.77, 2.28	0.31
Blood pressure, systolic (SBP)	mmHg	1.00	0.99, 1.02	0.55
Blood pressure, diastolic (DBP)	mmHg	1.02	0.997, 1.04	0.09
Blood pressure, mean	mmHg	1.01	0.99, 1.03	0.20
Ankle-brachial index	Index	1.49	0.26, 8.57	0.66
Arterial hypertension	Yes/no	1.23	0.71, 2.12	0.46
Arterial hypertension, stage	0–4	1.18	0.89, 1.57	0.25
Prevalence of chronic obstructive pulmonary disease	Yes/no	1.77	0.79, 3.95	0.17
Intake of blood lipid lowering medication	Yes/no	0.63	0.22, 1.75	0.37
Hearing loss	Hearing loss score (0–44)	1.00	0.98, 1.02	0.90
Depression Score	Depression score unit (range: − 4 to + 15)	0.96	0.89, 1.03	0.27
State-Trait Anxiety Inventory	State-Trait Anxiety Inventory Score (range: − 7 to 13)	0.99	0.92, 1.07	0.82
Manual dynamometry, right hand	dekaNewton	1.00	0.98, 1.03	0.86
Manual dynamometry, right hand	dekaNewton	1.01	0.98, 1.04	0.52

**Table 6:** Associations (binary univariate analysis) between the prevalence of angle-closure glaucoma and ocular parameters after adjusting for age in the Ural Eye and Medical Study.

Parameter	Interval	Odds Ratio (OR)	95% confidence interval of OR	*P* value
Best corrected visual acuity	logMAR	0.88	0.42, 1.86	0.74
Refractive error, spherical equivalent	Diopters	1.04	0.93, 1.18	0.49
Refractive error, cylindrical value	Diopters	0.89	0.68, 1.16	0.40
Axial length	mm	0.79	0.61, 1.02	0.07
Corneal refractive power	Diopters	0.91	0.78, 1.07	0.25
Central corneal thickness	µm	1.01	1.005, 1.02	0.001
Corneal volume	mm^3^	1.05	0.98, 1.12	0.16
Anterior chamber depth	mm	0.07	0.03, 0.13	< 0.001
Anterior chamber volume	µL	0.97	0.96, 0.98	< 0.001
Anterior chamber angle	Degree	0.83	0.80, 0.87	< 0.001
Lens thickness	mm	2.11	1.11, 4.01	0.02
Myopic maculopathy	Yes/no	0.00	0.00	1.00
Myopic maculopathy	Stage	0.51	0.15, 1.75	0.29
Nuclear cataract degree	Grade	0.79	0.58, 1.06	0.11
Nuclear cataract, presence	Yes/no	0.69	0.38, 1.24	0.21
Cortical cataract, degree	Percentage	1.00	0.99, 1.02	0.81
Cortical cataract, presence	Yes/no	0.51	0.24, 1.06	0.07
Subcapsular cataract, degree	Percentage	0.08	0.00	1.00
Subcapsular cataract, presence	Yes/no	0.00	0.00	1.00
Fundus tessellation, macula region	Grade	0.77	0.53, 1.12	0.17
Fundus tessellation, peripapillary region	Grade	0.97	0.73, 1.29	0.84
Intraocular pressure (mmHg)	mmHg	1.31	1.25, 1.37	< 0.001
Pseudoexfoliation of the lens, presence	Yes/no	2.07	0.92, 4.66	0.08
Pseudoexfoliation of the lens, grade	Grade	1.18	0.96, 1.46	0.12
Retinal thickness (total), fovea	µm	1.00	1.00, 1.00	0.34
Retinal thickness (total), 300 µm temporal to the fovea	µm	1.00	1.00, 1.01	0.98
Retinal thickness (total), 300 µm nasal to the fovea	µm	1.00	0.99, 1.00	0.99
Retinal nerve fiber layer thickness	µm	0.97	0.96, 0.98	< 0.001
Diabetic retinopathy	Yes/no	2.60	0.61, 11.1	0.20
Diabetic retinopathy, ETDRS grading	Scale	1.02	0.97, 1.07	0.48

The prevalence of angle-closure glaucoma was not significantly associated with the systemic parameters of gender, urban region of habitation, level of education, physical activity score, family status (married versus unmarried), religion (Muslim versus non-Muslim), body height, body weight, bod mass index, waist-hip ratio, history of angina pectoris, arthritis, cancer, cardiovascular diseases including stroke, dementia, diarrhea, heart attack, previous falls, iron-deficiency anemia, episodes with low blood pressure and hospitalization, osteoarthritis, injuries other than bone fractures, backache, neck pain, thoracic spine pain, skin disease, thyroid disorder, episodes of unconsciousness and menopause (age of any last bleeding; age of last regular bleeding). It was neither associated with the serum concentrations of alanine aminotransferase, aspartate aminotransferase, bilirubin, high-density lipoproteins, low-density lipoproteins, triglycerides, cholesterol, rheumatoid factor, glucose, creatinine, urea, residual nitrogen and hemoglobin, INR (international normalization ratio), the erythrocyte count, leucocyte count, intake of blood lipid lowering medication, the prevalences of diabetes mellitus, anemia and arterial hypertension, the stage of arterial hypertension, State-Trait Anxiety Inventory (STAI) score, diet parameters such as number of meals taken, number of days with fruit intake or vegetable intake, type of oil used for cooking, amount of food contained whole grain, amount of self-reported salt intake, and the grade of processing of meat (weak/medium/well done), systolic, diastolic and mean blood pressure, the ankle-brachial index, prevalence of arterial hypertension, current smoker, daily smoking, smoked package years, any alcohol consumed, hearing loss total score, depression score, and manual dynamometry. Nor was the prevalence of angle-closure glaucoma associated with the ocular parameters of refractive error, corneal refractive power, retinal thickness (total) in the fovea, prevalence and grade of pseudoexfoliation of the lens, prevalence and stage of nuclear cataract, cortical cataract and subcapsular cataract, prevalence and stage of diabetic retinopathy, prevalence and stage of myopic maculopathy, and lower BCVA.

In the multivariable analysis, we dropped due to collinearity the parameters of anterior chamber volume (VIF: 5.4) and anterior chamber depth (VIF: 2.2). Due to a lack of statistical significance, we dropped the parameters of central corneal thickness (*P* = 0.70), history of tumbling (*P* = 0.92), erythrocyte sedimentation rate (*P* = 0.29), ethnicity (*P* = 0.20), axial length (*P* = 0.70), and lens thickness (*P* = 0.09). In the final model, a higher prevalence of angle-closure glaucoma was associated with older age (OR 1.07; 95% CI 1.03, 1.11; *P* < 0.001), narrower anterior chamber angle (OR 0.81; 95% CI 0.77, 0.86; *P* < 0.001), higher intraocular pressure (OR 1.30; 95% CI 1.23, 1.38; *P* < 0.001), and thinner peripapillary retinal nerve fiber layer thickness (OR 0.97; 95% CI 0.95, 0.98; *P* < 0.001).

Among 184 individuals with moderate to severe vision impairment (MSVI) (defined as BCVA < 6/18 but ≥ 3/60 inclusive in the better eye or in binocular viewing), MSVI was due to glaucoma in nine individuals (4.9%; 95% CI 1.8, 8.1), among them seven individuals with open-angle glaucoma and two individuals with angle-closure glaucoma. Glaucoma (i.e., angle-closure glaucoma) was the cause for blindness (BCVA < 3/60) in one individual (9.1%) out of 11 blind individuals.

## Discussion

In our population-based study population, the prevalence of glaucoma was 4.4%, with open-angle glaucoma (3.2%) having a higher prevalence than angle-closure glaucoma (1.2%). Glaucoma prevalence increased from 0.6% in the age group of 40 to < 45 years to 20.0% in the age group of 80 + years. Factors associated with a higher prevalence of open-angle glaucoma were older age, longer axial length, higher IOP, higher stage of lens pseudoexfoliation, lower diastolic blood pressure and thinner peripapillary retinal nerve fiber layer thickness, or higher estimated trans-lamina cribrosa pressure difference, if age, IOP and blood pressure were dropped. Higher ACG prevalence correlated with older age, narrower anterior chamber angle, higher IOP and thinner RNFL. Among the 246 patients with glaucoma, 80 (32.5%) patients were on glaucoma therapy. Glaucoma caused MSVI in nine (4.9%) out of 184 individuals with MSVI (open-angle glaucoma, n = 7; angle-closure glaucoma, n = 2), and blindness in one individual (9.1%) out of 11 blind individuals. The single IOP measurement was ≤ 21 mmHg in 167 (67.9%) individuals with glaucoma.

The findings on the prevalence of glaucoma of 4.4% and of open-angle glaucoma of 3.2% in our study population agree with the observations made in some, and differ from the observations made in other, previous investigations. In the preceding studies, the prevalence of open-angle glaucoma varied between 0.5% in a rural population in Mongolia, 1.6% in a Singaporean urban population, 1.6% in an Indian urban population, and 1.7%, 2.1%, 2.7%, 3.0%, 3.0%, 3.8%, 5.7%, and 7.0% in populations from Melbourne, Australia, United States, South Africa, Sydney, East Africa, South China (Liwan District, Guangzhou), Central Sweden, and Barbados, to mention only few examples^20–47^. The glaucoma prevalence of 4.4% and prevalence of open-angle glaucoma of 3.2% in the Ural Eye and Medical Study was similar to the glaucoma prevalence of 3.7% reported in the Liwan Eye Study in Guangzhou/South China and of 3.8% as found in the Beijing Eye Study^22,46^. Similar glaucoma prevalences were also reported from Japan, India, and Singapore, while the glaucoma prevalence was higher in Sub-Saharan African countries. A meta-analysis analyzing data from 50 population-based studies found a global prevalence of glaucoma of 3.54% (95% credible intervals (CrI): 2.09–5.82) for the population aged 40–80 years^47^. In that analysis, the prevalence of open-angle glaucoma was the highest in Africa (4.20%; 95% CrI: 2.08–7.35), and the prevalence of angle-closure glaucoma was the highest in Asia (1.09%; 95% CrI: 0.43–2.32)^42^. Reasons for the discrepancies between the studies in the prevalence of the glaucomas may be ethnic differences, with a relatively high glaucoma prevalence in Sub-Saharan Africa, and differences in the examination technique and glaucoma definitions^17^.

The associations of the prevalence of the glaucomas with other ocular and general parameters as found in our study agrees with most of the previous investigations on different study populations^20–46^. It also includes the correlation between a higher prevalence of open-angle glaucoma and a longer axial length^46,48^. In agreement with previous studies and partially in contrast to some hospital-based studies, the prevalence of open-angle glaucoma was not related with central corneal thickness in the multivariable analysis^49^.

In our study population, the ratio of the prevalence of open-angle glaucoma to primary angle-closure glaucoma of 3.2–1.2% or 2.7 to 1 was similar to the findings in other population-based investigations such as the Beijing Eye Study with a ratio of 2.6–1^46^. In other studies different ratios were found such as in the South China Liwan Eye Study a ratio of 2.1–1.5% (or 1.4:1), the Japanese Tajimi Study with a ratio of 3.9–0.6% (or 6.5:1), the Singapore Malay Eye Study with a ratio of 2.5% to 0.1% (or 25:1),and the Tanjong Pagar study with a ratio of 2.4% to 0.8% (3:1)^21,22,45,50,51^. Reasons for differences between the studies and study populations may be differences in the definition of the glaucomas, ethnically associated differences in the anterior chamber anatomy, and differences in the prevalence of pseudophakia/aphakia.

Glaucoma caused MSVI in 4.9% of all individuals with MSVI and blindness in 9% of the blind individuals. These figures were comparable with the figures found in other countries. In the meta-analysis on the worldwide prevalence of MSVI and blindness by the Flaxman and colleagues, 8.49% (80% UI: 2.99–15.66) of all cases with MSVI and 2.5% (80% UI: 0.62–4.03) of all cases with blindness were caused by glaucoma^1^. Interestingly, seven out of the 9 individuals with glaucoma-related MSVI had open-angle glaucoma, while other studies reported that the prevalence of glaucoma-related blindness was higher in individuals with angle-closure glaucoma than in individuals with open-angle glaucoma^46^. The only participants with glaucoma-related blindness in our study population had angle-closure glaucoma.

Among the 246 patients with glaucoma, the single IOP measurement was ≤ 21 mmHg in 167 (67.9%) individuals. This figure was similar to a fraction of 70% of the glaucomatous eyes in the Beijing Eye Study with glaucomatous optic neuropathy and an IOP of ≤ 21 mmHg^52^. It shows the limited value of a single IOP measurement as screening method to detect glaucoma. Simultaneously, with only one third of the glaucomatous patients being on glaucoma therapy, screening measures, in particular on risk populations, should be developed and applied to reduce the risk of glaucoma-related blindness.

There are limitations of our investigation. First, we differentiated between open-angle glaucoma and angle-closure glaucoma by anterior segment imaging and did not perform gonioscopy. Since a closed anterior chamber angle can appear upon imaging as a falsely open angle, the prevalence of angle-closure glaucoma might have been underestimated. Second, we measured the IOP by non-contact tonometry instead of applanation tonometry. Third, the group of individuals with information about the presence of glaucoma as compared with the group of individuals without assessment of the presence of glaucoma was significantly younger and showed a significantly higher proportion of women, while both groups did not differ significantly in axial length. The younger age in the individuals with glaucoma assessment may have led to an underestimation of the prevalence of glaucoma in the total study population. Fourth, we tested associations between the prevalence of glaucoma and a whole panoply of ocular and systemic parameters, what might have led to falsely high significances in the univariate analyses. In full recognition of this potential weakness, a subsequent multivariate analysis assessed these associations with additionally taking into account interdependencies between the independent parameters. If despite of it a Bonferroni correction was performed for the results of the multivariate analysis, the associations of the prevalence of open-angle glaucoma with all parameters, except for the diastolic blood pressure, and the associations of the prevalence of angle-closure glaucoma with all parameters remained significant. Fifth, information about other (more rare) types of secondary open-glaucoma other than pseudoexfoliative glaucoma, such as uveitic glaucoma, has not been available for statistical analysis. Sixth, the perimeter used in our study has not extensively been used in international scientific studies, and its parameters of perimetric defects may not be fully congruent with the perimetric indices of the internationally established perimeters of Octopus or Humphrey. The definition of glaucomatous optic neuropathy may thus not be fully comparable to the definition applied in other studies. Sixth, the comparison of the glaucoma prevalence between the various studies is hampered since the studies varied in the definitions of glaucoma used and the examination techniques applied. In addition, the overall prevalence of glaucoma in the various study populations depends on their age and sex structure, so that age-specific and sex-adjusted prevalence data should be the basis for the comparison. For many of the previous studies however, such data in tabulated form are not fully available (Table 2).

In conclusion, in this typical ethnically mixed population from Russia with an age of 40 + years, the prevalence of glaucoma was 4.4% and increased with age, longer axial length, higher intraocular pressure, lens pseudoexfoliation, and lower diastolic blood pressure. The glaucoma prevalence was comparable with figures from Caucasian and East Asian populations, and it was lower than the figures for Sub-Saharan African populations. The ratio of open-angle glaucoma to angle-closure glaucoma of 2.7:1 and the percentage of glaucoma-related MSVI on total MSVI and of glaucoma-related blindness on total blindness roughly agree with the findings obtained in other recent studies. Two thirds of glaucoma patients were not on therapy, and in two thirds of the glaucoma patients the single IOP reading was ≤ 21 mmHg.
